# Influence of root-bed size on the response of tobacco to elevated CO_2_ as mediated by cytokinins

**DOI:** 10.1093/aobpla/plu010

**Published:** 2014-03-17

**Authors:** Ulrike Schaz, Barbara Düll, Christiane Reinbothe, Erwin Beck

**Affiliations:** 1 Department of Plant Physiology, University of Bayreuth, Universitätsstrasse 30, 95440 Bayreuth, Germany; 2 Present address: Department of Anatomy and Cell Biology, University of Ulm, Albert-Einstein-Allee 11, D-89081 Ulm, Germany

**Keywords:** Biomass portioning, C/N ratio, cytokinins, elevated CO_2_, growth, root-bed volume, tobacco.

## Abstract

The extent of growth stimulation of C_3_ plants by elevated CO_2_ is modulated by environmental factors. Under optimized environmental conditions (high light, continuous water and nutrient supply, and others), we analysed the effect of an elevated CO_2_ atmosphere (700 ppm, EC) and the importance of root-bed size on the growth of tobacco. Biomass production was consistently higher under EC. However, the stimulation was overridden by root-bed volumes that restricted root growth. Maximum growth and biomass production were obtained at a root bed of 15 L at ambient and elevated CO_2_ concentrations. Starting with seed germination, the plants were strictly maintained under ambient or elevated CO_2_ until flowering. Thus, the well-known acclimation effect of growth to enhanced CO_2_ did not occur. The relative growth rates of EC plants exceeded those of ambient-CO_2_ plants only during the initial phases of germination and seedling establishment. This was sufficient for a persistently higher absolute biomass production by EC plants in non-limiting root-bed volumes. Both the size of the root bed and the CO_2_ concentration influenced the quantitative cytokinin patterns, particularly in the meristematic tissues of shoots, but to a smaller extent in stems, leaves and roots. In spite of the generally low cytokinin concentrations in roots, the amounts of cytokinins moving from the root to the shoot were substantially higher in high-CO_2_ plants. Because the cytokinin patterns of the (xylem) fluid in the stems did not match those of the shoot meristems, it is assumed that cytokinins as long-distance signals from the roots stimulate meristematic activity in the shoot apex and the sink leaves. Subsequently, the meristems are able to synthesize those phytohormones that are required for the cell cycle. Root-borne cytokinins entering the shoot appear to be one of the major control points for the integration of various environmental cues into one signal for optimized growth.

## Abstract (precis)

The effect of elevated CO_2_ on the growth of tobacco under high light (16 h), continuous water and nutrient supply was investigated. Biomass production depended strongly on the size of the root bed. Inhibition by a small root bed was higher at 700 than at 360 ppm CO_2_. Relative growth rates showed a head-start of the high-CO_2_ plants which gave rise to a persistently higher biomass production. Root-bed size and CO_2_ concentration were mirrored by the quantitative cytokinin patterns of the various plant parts. Amounts of the cytokinins moving from the root to the shoot were higher in high-CO_2_ plants.

## Introduction

Current CO_2_ scenarios (IPCC 2013) predict CO_2_ concentrations of 700 ppm at the end of the 21st century (Prentice *et al.* 2001). Such an increase will affect natural and agricultural ecosystems as ‘green CO_2_ sinks’ (Drake *et al.* 1997; Krupa 2003; Lindroth 2010; Hikosaka *et al.* 2011) and thus may have far-reaching economic and political consequences. Knowledge of the reaction of plants to elevated CO_2_ concentrations is essential for assessing the potential capacity of green CO_2_ sinks.

In numerous laboratory and field studies, different responses of plants to elevated CO_2_ concentrations have been observed (Makino and Mae 1999; Poorter and Navas 2003; Long *et al.* 2006; for a review see Körner 2006). Apart from the CO_2_ concentration itself, these effects were modulated by a variety of factors, e.g. light intensity, temperature, and water and nutrient (especially nitrogen) supply (McConnaughay *et al.* 1993; Stitt and Krapp 1999; Ainsworth and Long 2005; Johnsen 2006; Reich *et al.* 2006; for a review see Kant *et al.* 2012). Differences in the reactions of plants to elevated CO_2_ depend also on the experimental approach: chamber studies with plants grown in pots versus field studies using free-air concentration enrichment (Long *et al.* 2006; Ainsworth *et al.* 2008).

For C_3_ plants, an initial and transitory increase of the rates of photosynthesis and plant growth was generally observed upon exposure to elevated CO_2_ concentrations, a phenomenon that has been termed ‘acclimation’ (Long *et al.* 2004). Acclimation to elevated CO_2_ results from a decrease of Rubisco activity, for which several explanations have been presented, in particular an internal feedback mechanism termed ‘sink limitation’. If production of photosynthates in the leaves exceeds utilization by the various sink activities of a plant, e.g. root and shoot growth, export via the phloem decreases and carbohydrates accumulate as starch in the chloroplasts (Thomas and Strain 1991). This in turn inhibits chloroplastic metabolism, in particular photosynthesis (Stitt 1991; Paul and Foyer 2001). Thus, the response of a plant to elevated CO_2_ should be governed by the activities of its sinks (Reekie *et al.* 1998). High sink activities can only be expected under optimal growth conditions, which hardly occur in nature. Under optimal light, high CO_2_ concentration and sufficient water supply, macronutrients may readily become limiting. Deterioration of the plant's nutrient status (in particular nitrogen) due to an attenuation of nutrient uptake might affect growth and thus sink activities, and in turn cause ‘acclimation’ to elevated CO_2_ (Makino *et al.* 1997; Nakano *et al.* 1997; Sicher and Bunce 1997; Curtis *et al.* 2000). Decreasing nutrient uptake could result from a declining nutrient concentration in the root bed (Geiger *et al.* 1999; Stitt and Krapp 1999) or by a dwindling uptake capacity of the roots (Thomas and Strain 1991). Both reasons often coincide in pot experiments with too small root-bed volumes that restrict root growth (Arp 1991; Berntson *et al.* 1993; McConnaughay *et al.* 1993; Rabha and Uprety 1998; Zhu *et al.* 2006; Yang *et al.* 2007, 2010). This is important as elevated CO_2_ concentrations stimulate root growth, in particular fine root production (Norby *et al.* 2004; Stiling *et al.* 2013), and thus a small root-bed size should restrict root growth much more at elevated than at ambient CO_2_ concentrations.

Both increased root growth in response to elevated CO_2_ on the one hand and limited root growth by root-bed size on the other hand must be balanced by the plant. It must be able to recognize the ‘nutrient status’ of the root and of the shoot and ‘translate’ this information into a growth response (Berntson *et al.* 1993; McConnaughay *et al.* 1993). To adapt growth to the resource status, plants rely on specific long-distance growth signals that mediate the communication between the root and the shoot. At least with respect to nitrogen as the most important macronutrient, translation of the ‘resource status’ of the root into a long-distance signal—cytokinins—has been shown (Beck and Wagner 1994; Beck 1999; Yong *et al.* 2000; Miyawaki *et al.* 2004).

Cytokinins are mitogenic signals that control the cell cycle (Francis and Sorrell 2001; Hartig and Beck 2005; Harashima and Schnittger 2010; Dudits *et al.* 2011) and thus activity of meristems. Reduced cytokinin contents in transgenic tobacco and *Arabidopsis* plants resulted in slow-growing, stunted shoots with small leaves but an enhanced root system (Werner *et al.* 2001, 2003, 2008; Yang *et al.* 2003).

Of course, cytokinins are not the only long-distance and cellular signals that adapt plant growth to its resource status, but they may be the most prominent ones. Evidence is increasing that it is not the cytokinin load exported from the root to the shoot which directly controls the activity of the shoot meristems, but that the meristematic cells convert such exogenous into intracellular cytokinin signals, which however are also under the control of other signals (Bürkle *et al.* 2003; Motyka *et al.* 2003). Bishopp *et al.* (2011) showed that cytokinins moving from the shoot to the root via the phloem play a role in vascular patterning of the root apex.

In the present study, the growth response of tobacco plants to an elevated CO_2_ concentration was analysed under controlled conditions (optimal water and nutrient supply as well as light conditions). In contrast to similar studies reported so far, an optimized nutrient solution was continuously flushed, day and night, through the root beds of pure quartz sand, keeping the nutrient concentration around the roots constant and thus avoiding effects of changing nutrient concentrations.

With that approach, especially the improved nutrient supply, the following hypotheses were examined: (i) under our conditions, the size of the root bed is the crucial factor that controls growth of the plant under ambient as well as elevated CO_2_ concentrations, and therefore (ii) acclimation of plants to elevated CO_2_ can be prevented by a sufficiently large root bed. (iii) We further hypothesize that cytokinins are involved in growth stimulation under conditions where acclimation is avoided. To this end, cytokinins in different plant parts and in the xylem sap were quantified comparing tobacco plants grown under ambient and elevated CO_2_ and cultivated in growth limiting and non-limiting root beds.

## Methods

### Plant growth and experimental setup

#### Plant growth

*Nicotiana tabacum* cv. *Samsun* was grown in two accurately controlled walk-in climate chambers (York International, volume 11.5 m^3^). The light period of 14 h at 26 °C and 70 % relative humidity was followed by a 10-h dark period at 19 °C and 60 % relative humidity. The light intensity at the top of the pots was 700 µmol photons m^−2^ s^−1^ (MT 400 DL/BH E-40 lamps; Iwasaki Electric Co*.*, Tokyo, Japan). The dark phase included 30 min ‘dawn’ and ‘dusk’ with increasing or decreasing light intensities. The CO_2_ concentration in the control chamber was 360 ppm (atmospheric ambient concentration, ‘AC’) whereas in the other chamber a permanent CO_2_ concentration of 700 ppm CO_2_ (elevated, ‘EC’) was provided (the CO_2_ concentration was automatically controlled with a gas exchange analyser, BINOS; Leybold-Heraeus).

Tobacco seeds were germinated in transparent boxes (Phytotray II; Sigma) on agar in the respective climate chambers at 360 or 700 ppm CO_2_. The medium for germination contained 0.5 % (w/v) inorganic Murashige–Skoog medium, pH 5.7 (Murashige and Skoog 1962), and 0.5 % (w/v) agar to which a small quantity of active carbon powder was added to avoid infestation by fungi. After sowing, the boxes were closed and covered with a net (mesh size 8 mm) to mitigate illumination strength. After 1 week, the boxes were opened once per day to allow exchange with the atmosphere of the climate chamber.

Twelve to 15 days after sowing, when the cotyledons had completely unfolded, the seedlings were transferred to sand culture. The root bed consisted of pure quartz sand (grain size 0.7–1.2 mm) that—after washing with one pot volume of deionized water—was rinsed with half of the pot volume of diluted nutrient solution (nutrient solution : water = 1 : 3). The nutrient solution contained 3 mM K_2_HPO_4_, 4 mM KNO_3_, 4 mM Mg(NO_3_)_2_, 2 mM MgSO_4_, 4 mM CaSO_4_, 0.02 mM Fe-EDTA, 50 µM KCl, 20 µM H_3_BO_4_, 2 µM MnSO_4_, 2 µM ZnSO_4_, 0.5 µM CuSO_4_ and 0.5 µM MoO_3_ (pH 6.0, adjusted with H_2_SO_4_; Dertinger *et al.* 2003). After transfer to the sand culture, the pots were initially covered with cellophane for maintaining high air humidity.

Two days later, the cellophane was replaced by a shading net, under which the plantlets were kept for 12 days. The root bed was continuously percolated with nutrient solution with a pump (IPC-N; Ismatec Laboratoriumstechnik GmbH, Wertheim, Germany). To maintain the nutrient concentration around the roots constant, the root bed was percolated with 300 mL of nutrient solution per 1 L of root-bed volume and day. During the first 2 weeks, the plantlets were supplied with a half concentrated nutrient solution. Thereafter, undiluted nutrient solution was supplied. To provide equal growth conditions for all plants, the pots were turned around and their places in the climate chambers were exchanged twice a week.

#### Experimental setup

For close comparison, tobacco plants were cultivated at the same time under the two CO_2_ concentrations in pots of 1, 5, 10, 15 and 20 L volume until an age of 61 days. Five replicates were grown per pot volume (triplicates in the 20-L pot experiment). A more detailed time kinetics of growth and biomass partitioning under AC and EC was made with the plants in the 15-L pots.

Biomass (dry weights) and relative growth rates (RGRs) of the plantlets were investigated at the ages of 1, 2, 3, 4, 5, 6, 7, 8, 9, 12 and 15 days after sowing in 5-fold replicates samples of 10 plants each. The older plants were examined in triplicate at 21, 28, 35, 42, 48 and 61 days after sowing. The leaves were numbered from the bottom to the top, beginning with the first leaf following the cotyledons. For comparing parameters of older plants, the parameter ‘physiological age’ was used, which was defined by the number of leaves and the area of the biggest leaf (see Table 1).

**Table 1.** Number of leaves, leaf length, leaf width and plant age of *N. tabacum* cv. *Samsun* grown at 360 and 700 ppm CO_2_, respectively, in 15-L sand culture. The leaves were counted from bottom to top. Leaf length and leaf width of the biggest leaf per plant were determined.

Number of leaves		Biggest leaf (leaf number)	Leaf length × leaf width (mm × mm)		Plant age (days)
360 ppm CO_2_	700 ppm CO_2_		360 ppm CO_2_	700 ppm CO_2_	
11	12	7	105 × 80	130 × 100	28
15	16	8	170 × 125	195 × 145	35
22	23	9	210 × 180	250 × 200	42
25	25	10	240 × 200	280 × 220	49

Xylem sap was collected at two sites of 35-day-old plants: at the base of the stem just above leaf 4 and at the petiole of a source leaf (leaf 8). The effect of transpiration (measured with a porometer) was compensated by pressurizing the root with nitrogen in a root pressure chamber (Passioura 1980). Before cutting, the pressure in the root chamber was slightly higher than necessary for compensating the transpiration to avoid embolism. Immediately after cutting, the flow rate was adjusted by reducing the pressure on the root. The first 100 µL of the sap samples were discarded. Xylem sap was subsequently collected for 1 h and stored at −20 °C until cytokinin analysis.

### Measurements

Leaf area was calculated by the following equation:

$$\hbox{leaf}\;\hbox{area} = 0.74 \times \hbox{length} \times \hbox{width}$$

**[see Supporting Information]**.

Plants were consistently harvested in the middle of the light period. Dry weights were determined after drying the material at 80 °C to constant weight. Relative growth rate was calculated by the equation

$$R = \ln ({\hbox{dw}_1 /\hbox{dw}_{\rm 0} } )/({t_1 - t_0 } )$$

where dw_1_ is the dry weight of the plant at the time of measurement *t*_1_ and dw_0_ the dry weight at the starting time *t*_0_.

Carbon and nitrogen contents were determined with an element analyser (CHN-O-Rapid; Elementar Analysesysteme GmbH, Hanau, Germany). The dried material was homogenized in a ball mill (Schwingmühle MM2000; Retsch GmbH & Co. KG, Haan, Germany) and aliquots of the powder were analysed. Acetanilide (71.09 % carbon and 10.36 % nitrogen) was used as the standard.

For cytokinin analysis, the procedure of Wagner and Beck (1993) modified by Hartig and Beck (2005) was followed. This method is more complex than the currently used detection by liquid chromatography-mass spectrometry. It has carefully been elaborated for several plant tissues—among others for tobacco—and for analysis of xylem sap using cross-reactivity of the diverse antibodies as internal control **[see****Supporting Information****]**. It separates the cytokinins (also the aromatic ones), its nucleotides and glucosides and thus provides a comprehensive view of the entire cytokinin pattern. Plant material, as shown in **Supporting Information**, was extracted with 80 % aqueous methanol in the cold and the extracts were purified by reverse-phase (RP) column chromatography (Bakerbond spe™ octadecyl (C_18_) disposable extraction column; J. T. Baker, Deventer, Holland) with 80 % methanol. The cytokinins were fractionated by anion exchange chromatography on a DEAE Sephadex™ A-25 column (Amersham Pharmacia Biotech AB, Uppsala, Sweden) into two fractions, using 40 mM NH_4_OAc buffer (pH 6.5) as the eluent: fraction (i) containing the bases (*t*-Z, DHZ, IP) + ribosides (*t*-ZR, DHZR, IPA) + glucosides (Z9G, ZOG, ZROG, DHZ9G, DHZOG, DHZROG) and fraction (ii) with the nucleotides (ZN, DHZN, IPN). To collect fraction (i), a 1.5-mL RP cartridge (Sep-Pak^®^; Waters Corporation, Milford, MA, USA) was coupled to the anion exchange column. The nucleotides were subsequently eluted from the anion exchanger with 6 % formic acid (v/v) into another RP cartridge from which they were collected with 80 % aqueous methanol. The individual cytokinins were separated by preparative RP-HPLC (column: 250 mm long, 4.6 mm i.d., 5 µm Hypersil^®^ ODS; Muderer & Wochele, Berlin, Germany) and collected in 36 fractions. Elution was carried out with a gradient of acetonitrile as the non-polar phase and 0.1 % (v/v) aqueous triethylammonium acetate in bi-distilled water, pH 6, as the polar phase (for the protocol, see Wagner and Beck 1993). The different cytokinins were quantified by competitive enzyme-linked immunosorbent assay (ELISA) using three phosphatase-coupled antibodies (anti-*t*-ZR, anti-DHZR and anti-IPA; Wagner and Beck 1993). The bases and 9-glucosides could be determined due to their cross-reactivity with the three antibodies (Weiler and Zenk 1976; Wagner and Beck 1993). The *O*-glucosides, which did not show cross-reactivity, were quantified as free bases or ribosides after removal of the *O*-glucosyl residues with β-glucosidase (Wang *et al.* 1977). The nucleotides were analysed as ribosides after dephosphorylation with alkaline phosphatase followed by ELISA.

The yield of the whole procedure determined with internal standards was more than 90 % and the reliability of the ELISA was examined according to Pengelly (1986) and Crozier *et al.* (1986). The sources of the chemicals and the standard substances as well as the preparation of the standard solutions have been described in detail by Wagner and Beck (1993).

### Statistics

The approach encompassed two variables: two CO_2_ concentrations and five different volumes of the root beds. Assessment of the effects of root-bed size on plant growth required comparison under identical environmental conditions, i.e. five root-bed variants (with replications) each under ambient and elevated CO_2_, respectively. Owing to spatial limitations, the entire setup encompassing the subprojects ‘Age-dependence of the effect of the CO_2_ concentration on the growth of tobacco plants’ and ‘Cytokinin patterns in tobacco plants grown under limiting/non-limiting root-bed conditions and at ambient or elevated CO_2_’ could be performed only once. Therefore, the question of pseudoreplication (Hurlbert 1984; Waller *et al.* 2013) arises. Since we had only two walk-in climate chambers with sophisticated control of environmental parameters, we cannot principally rule out pseudoreplication of the CO_2_ effect with our sample and replication sets. However, due to the fact that for a given root-bed volume all environmental factors including the composition of the root bed were carefully controlled and maintained while only the CO_2_ concentration was different, we think that the presented results are trustworthy. Furthermore, the CO_2_ concentrations were addressed as ‘ambient’ and ‘elevated’ and correlations with the absolute values of these concentrations were not established, and thus the danger of erroneous results due to pseudoreplication should be low.

Statistical analyses were carried out with SigmaStat (Systat Software GmbH, Germany). The number of replicates varied between 3 and 30 (and between 2 and 5 for cytokinin determination), as shown in the figures. As the data were not Gaussian distributed and the variances were not homogeneous (as tested by Kolmogorov–Smirnov's test), statistical differences between data from AC and EC plants (weights, shoot–root and shoot–leaf ratios, the leaves' carbon and nitrogen contents and their ratios, and the concentration of cytokinins) were examined using the Wilcoxon–Mann–Whitney test. Statistically significant results were indicated in the figures by asterisks: 0 < *P* ≤ 0.05*, 0.05 < *P* ≤ 0.01** and 0.01 < *P* ≤ 0.001***.

## Results

### Influence of root-bed volume and CO_2_ concentration on plant growth rates, morphology, biomass production and carbon/nitrogen status

**Figure 1.**
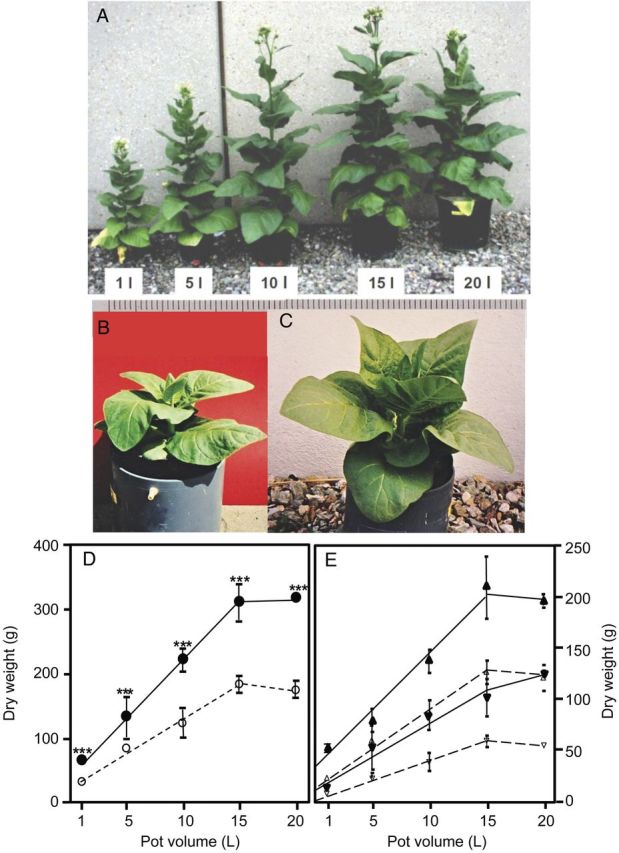
(A) Sixty-one-day-old tobacco plants grown in different pot volumes at 700 ppm CO_2_, (B) 35-day-old tobacco plants grown at 350 ppm or (C) at 700 ppm CO_2_. (D) Dry weights of entire 61-day-old tobacco plants grown at 360 ppm (open circles) or 700 ppm CO_2_ (closed circles), respectively (means of *n* = 5 and *n* = 3 for the 20-L pot and standard deviation), and (E) shoot (open upright triangle, black upright triangle) and root (open inverted triangle, black inverted triangle) dry weights of the 61-day-old tobacco plants grown in the indicated pot volumes under ambient (360 ppm; open upright triangle, open inverted triangle) and elevated (700 ppm; black upright triangle, black inverted triangle) CO_2_ concentrations. Mean values ± standard deviation are presented (*n* = 5 for 1-, 5-, 10- and 15-L pots, *n* = 3 for 20-L pots). Asterisks show statistical significances between plants grown under the two CO_2_ concentrations.

Tobacco plants were grown under ambient or elevated CO_2_ concentration in root-bed volumes ranging from 1 to 20 L. Figure 1A illustrates that the size of the plants and the leaf areas of the 61-day-old plants increased with the pot size of up to 15 L. The dry weights of plants increased linearly with the pot size at both CO_2_ concentrations (Fig. 1D). At the same pot size, plants grown at elevated CO_2_ (Fig. 1C) were larger and had higher dry weights, but had only one leaf more than plants grown at ambient CO_2_ (Fig. 1B). The pot size of 15 L was optimal for growth at both CO_2_ concentrations and a further increase to 20 L had no further effect on the size and biomass production.

Depending on their position on the stem and their physiological role as source, expanding or sink leaves, the net CO_2_ uptake rates of the individual leaves differed. While the CO_2_ concentration had no influence on net CO_2_ uptake by older, fully expanded leaves, younger, still growing leaves showed higher assimilation rates under elevated CO_2_ **[see Supporting Information]**.

In order to investigate the influence of root-bed size at ambient and elevated CO_2_ on biomass partitioning, the shoot and root dry weights were determined (Fig. 1E). All plants allocated more biomass to the shoot than to the root.

**Figure 2.**
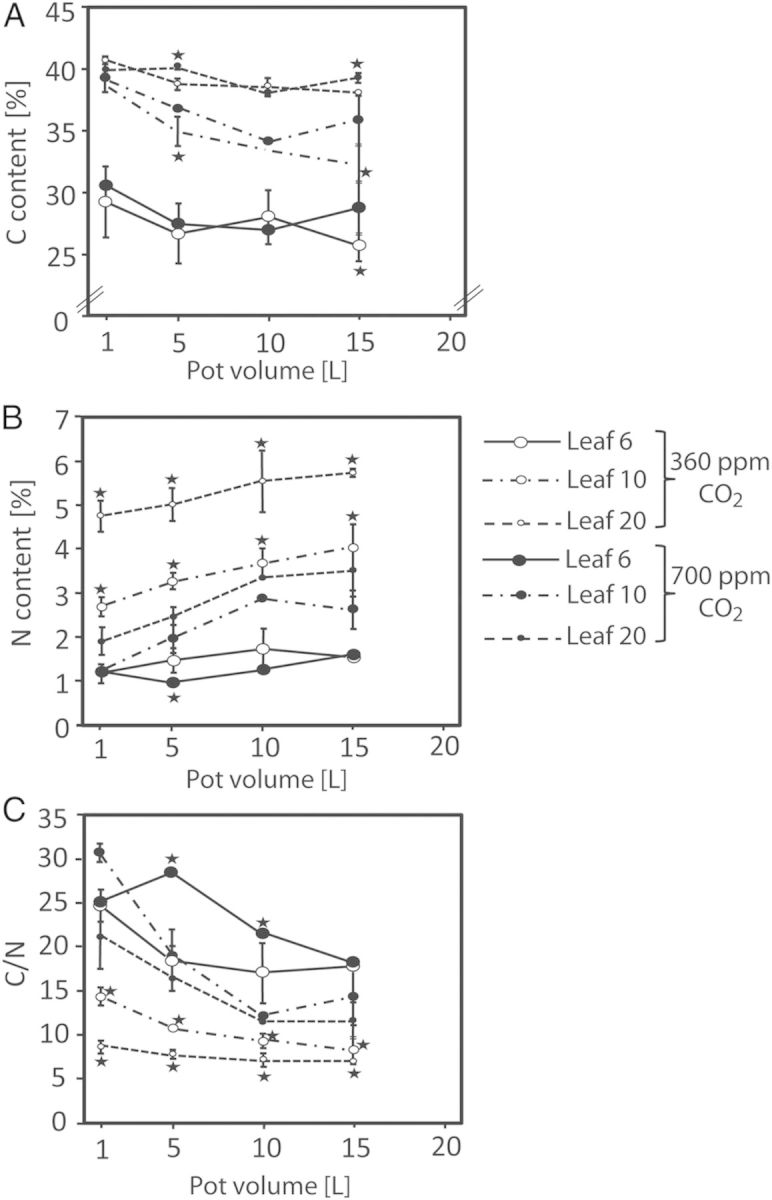
(A and B) Carbon and nitrogen content (%), and (C) C/N ratio of leaves 6, 10 and 20 (numbered from the bottom) of 61-day-old tobacco plants grown in different pot volumes under ambient (360 ppm) and elevated (700 ppm) CO_2_ concentration (means of *n* = 5 and standard deviation). Asterisks as in Fig. 1.

Alleviating root growth limitation by increasing the volume of the root bed and promoting root growth by elevated CO_2_ should result in enhanced nutrient uptake from the continuously supplied nutrients. This was examined by the carbon/nitrogen (C/N) ratio of the plants, of which three leaves (leaves 6, mature; 10, still expanding; and 20, young) were selected for determination of their carbon and nitrogen contents (Fig. 2A–C). Mainly due to the enhanced nitrogen content, the C/N ratios declined with increasing root-bed volumes, demonstrating the expected effect (Fig. 2C). Leaves of high-CO_2_ plants had consistently higher C/N ratios than those of ambient-CO_2_ plants. At both CO_2_ conditions, the C/N ratios increased with leaf age.

### Cytokinin patterns in tobacco plants grown under limiting/non-limiting root-bed conditions and at ambient or elevated CO_2_

Cytokinins are known as signals from the root to the shoot, which respond to the supply of nutrients to and their concentrations in the root. However, also the meristems of the shoot are capable of cytokinin production and the fully expanded leaves, which upon transpiration receive cytokinins via the xylem sap, must be able to metabolize these phytohormones or reload it to the phloem (Beck 1999; Yong *et al.* 2000). For a better understanding of the effect of root growth restriction and the response to the CO_2_ concentration on the cytokinin signal, we investigated (i) the cytokinin patterns of tobacco plants grown in a restricted or optimal root-bed volume under ambient or elevated CO_2_ and (ii) examined the effect of the CO_2_ concentration separately in an experiment designed for an assessment of the significance of cytokinins as a root signal.

#### Cytokinin patterns in organs of tobacco plants

**Figure 3.**
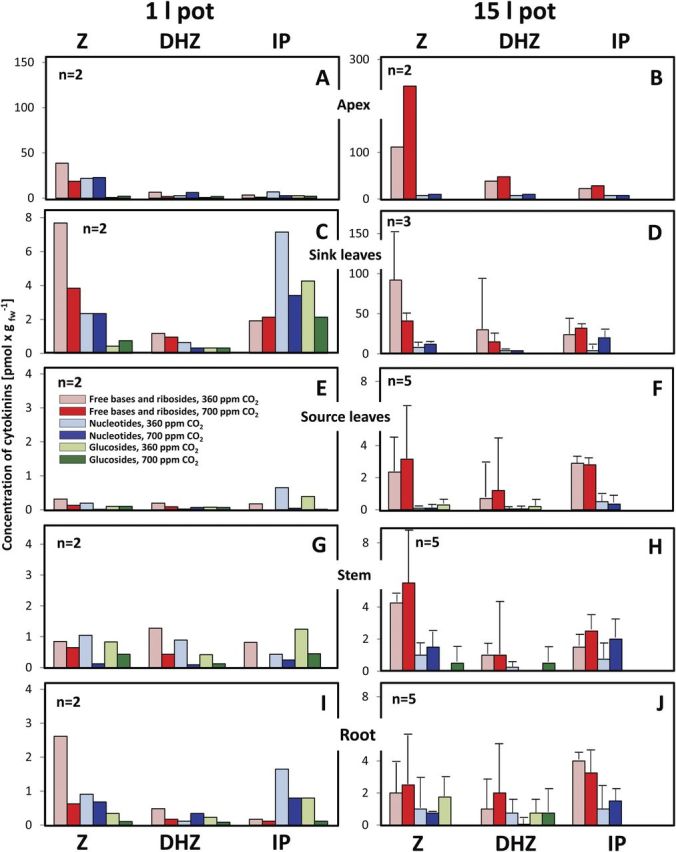
Concentrations of cytokinins in the apices (A, B), sink leaves (C, D), source leaves (E, F), stems (G, H) and roots (I, J) of 42-day-old tobacco plants grown in 1-L sand (A, C, E, G, I) or 15-L sand culture (B, D, F, H, J) under 360 and 700 ppm CO_2_, respectively (means of at least two independent experiments ± standard deviation). The concentrations of the free bases and ribosides as well as of the glucosides of each of the measured families were combined. Z, members of the *t*-zeatin family; DHZ, members of the dihydrozeatin family; IP, members of the isopentenyladenine family.

Cytokinins of plants from 1- and 15-L pots that had been grown in the described climate chambers under 360 and 700 ppm CO_2_, respectively, were analysed (Fig. 3).

The age of 42 days was chosen, as it still represents the late phase of vegetative growth. For the sake of clarity, the concentrations of the so-called active cytokinins, namely the free bases and the ribosides of each of the three examined cytokinin families, were combined (Mok and Mok 2001). Nucleotides which by dephosphorylation can readily give rise to active cytokinin species as well as the inactive glucosylated species are shown separately.

A clear positive effect of root-bed size was observed in the concentrations of members of all three cytokinin families in the meristematic tissues of the shoot, namely the apices and the unfolding small leaves around the shoot apex (‘sink leaves’). On average, concentrations were up to 6-fold higher in meristems of plants from the 15-L pots, with extremes of 28- and 35-fold higher concentrations for individual cytokinin species (compare Fig. 3A and B and Fig. 3C and D). *trans*-Z and its derivatives were the most prominent cytokinin species in apices and sink leaves with concentrations up to 37 pmol g^−1^ fresh weight (*t*-zeatinriboside) in plants from 1-L pots (Fig. 3A) and up to 140 pmol g^−1^ fresh weight (*t*-zeatin) in those from 15-L pots (Fig. 3B). Concentrations of dihydrozeatin and isopentenyladenine and their derivatives were on average 4- to 5-fold lower. Cytokinin concentrations were much lower in mature leaves, stems and roots, and with one exception (*t*-zeatinriboside in the stems of plants from 15-L pots) out of more than 100 values lower than 2.5 pmol g^−1^ fresh weight. Differences between the cytokinin concentrations in mature leaves, stems and roots of plants from 1- and 15-L pots were small with a slightly higher concentration in plants from the larger pots. The concentrations in roots as the major cytokinin-producing organs were surprisingly low, whereby representatives of the isopentenyladenine group equalled those of the *t-*Z family. It should be mentioned, however, that the concentrations do not reflect the total amounts, which—due to the different biomasses—are less conclusive. Substantial concentrations of the inactive *O*- and *N*-glycosides of the *t*-zeatin and dihydrozeatin families were not found in any of the organs, irrespective of pot size or CO_2_ concentration. Also, a consistent effect of CO_2_ concentration on the cytokinin patterns could not be detected in spite of several significant differences in the concentrations of individual cytokinin species.

### Effect of CO_2_ concentration on cytokinin signal

While an increase of root-bed size had only a small positive effect on cytokinin concentrations in the roots, a strong effect was observed in the shoot apex and the sink leaves. Also, the difference between the cytokinin concentrations in ambient- and high-CO_2_ plants from the same root-bed volume was negligible in the roots but high in the meristematic tissues of the shoot. Thus, the question arises as to how cytokinin translocation from the roots by the xylem stream can control growth and development of the shoot. Using the root pressure chamber (Passioura 1980), we collected xylem fluid from 35-day-old tobacco plants grown in root beds of 10 L in the described climate chambers. The technical limitation in this experiment was the size and number of root pressure chambers. Xylem fluid from the entire stem was collected for 1 h at the natural flow rates above the fourth node and from comparable plants from the petiole of a mature leaf (leaf 8). The cytokinin patterns of these xylem fluids **[Supporting Information]** were compared with those of the roots and the corresponding mature leaves, respectively (Table 2). Multiplication of the cytokinin concentrations with the fresh weight of the investigated plant organs yielded the total amounts. Likewise, the flow rates of cytokinins were calculated from the concentrations in the xylem fluid and the respective measured transpiration rates. The cytokinin concentrations in the transpiration stream and the total amounts of transported cytokinins were higher in the high-CO_2_ plants than in the ambient-CO_2_ plants. The cytokinin patterns in the fluid from the base of the stem did not exactly match the patterns in the root where the concentrations and amounts of the *t*-zeatin group were significantly higher. Comparison of the cytokinin patterns of the xylem fluids from the petioles of mature leaf no. 8 with those of the fluids collected at the bottom of the stems showed two major differences: the concentrations of the cytokinins were considerably lower and the xylem fluid in the petiole appeared to be substantially depleted of the dihydrozeatin and the isopentenyladenine cytokinins **[Supporting Information]**. But the quantitative cytokinin patterns in the xylem fluid from the petioles from ambient- and high-CO_2_ plants were almost identical.

**Table 2.** Effect of CO_2_ concentration on the concentration of cytokinins (Cks; Z, members of the *t*-zeatin family; DHZ, members of the dihydrozeatin family; IP, members of the isopentenyladenine family) in mature leaves and roots as well as the xylem sap of petioles from mature leaves and stems (see explanations in the text for further details on the collection of xylem sap) from 35-day-old tobacco plants grown in root beds of 10 L. Units corresponding to italic values are indicated in italics.

	Cks in mature leaf (*pmol g FW^−1^*) (pmol leaf^−1^)			Transpiration (*nL cm^−2^ s^−1^*) (mL h^−1^ leaf^−1^)	Cks in xylem sap (*nM*) (pmol leaf^−1^ h^−1^)		
	Z	DHZ	IP		Z	DHZ	IP
360	*1*.*11*	*1*.*76*	*2*.*31*	*10.6* ± *1.6*	*0*.*89*	*0*.*44*	*0*.*42*
	4.91	7.80	10.23	5.51 ± 0.92	4.73	2.42	2.31
700	*0*.*78*	*1*.*17*	*1*.*51*	*8.04* ± *1.61*	*0*.*74*	*0*.*44*	*0*.*28*
	7.58	11.37	14.68	5.84 ± 1.11	4.32	2.57	1.63

	**Cks in the roots (*pmol g FW^−1^*) (pmol per root system)**			**Transpiration (mL h^−1^ shoot^−1^)**	**Cks in xylem sap (*nM*) (pmol shoot^−1^ h^−1^)**		
	Z	DHZ	IP		Z	DHZ	IP
360	*11*.*1*	*4*.*3*	*4*.*3*	15.2 ± 0.68	*4*.*5*	*3*.*3*	*4*.*3*
	160	62.1	62.5		68.2	49.9	64.6
700	*7*.*7*	*1*.*9*	*2*.*1*	14.5 ± 2.37	*6*.*2*	*3*.*8*	*4*.*7*
	193	47.3	53.3		90.0	55.2	67.4

### Age dependence of the effect of CO_2_ concentration on the growth of tobacco plants

**Figure 4.**
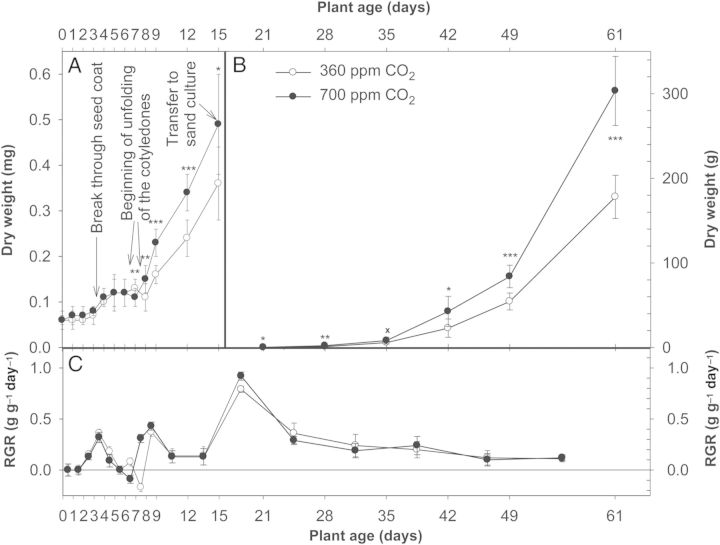
Increase of dry weights and the RGRs of tobacco plants after germination and growth under ambient (360 ppm) and elevated (700 ppm) CO_2_ concentration. (A) Dry weights (mg) of the seedlings after sowing (mean of *n* = 30 and standard deviation), (B) dry weights (g) of the tobacco plants after transfer to 15-L sand culture (mean of *n* = 5 and standard deviation) and (C) RGR (g g^−1^ day^−1^) calculated from the dry weights. Asterisks as in Fig. 2.

The results described so far revealed a growth-stimulating effect of elevated CO_2_ on the growth of tobacco plants. However, the question remained about the onset and age dependence of that effect. Germination, seedling development and growth of tobacco plants were therefore followed under ambient or elevated CO_2_ concentrations at a sufficiently large root bed (Fig. 4). Germination (on agar) started 3 days after sowing irrespective of the CO_2_ concentration (Fig. 4A). Under both CO_2_ concentrations, apparent RGRs increased from day 1 to day 4 mainly due to water uptake. Thereafter, RGR decreased to zero prior to unfolding of the cotyledons (Fig. 4C). After development of the cotyledons (days 6 and 7 under elevated CO_2_ and days 7 and 8 under ambient CO_2_), the seed coat was shed decreasing RGR to values below zero, which also indicated biomass loss by respiration. After the start of photosynthesis, dry weights and RGRs increased substantially, whereby the increase at elevated CO_2_ showed a head start of 1 day over that at ambient CO_2_. Already 8 days after sowing, seedlings grown under 700 ppm CO_2_ were significantly heavier than those grown under 360 ppm CO_2_. The higher initial RGR of high-CO_2_ seedlings, however, was caught up within 24 h by the ambient-CO_2_ plantlets, leading to equal RGRs from day 9 to day 13 after sowing. Nevertheless, the initially higher RGR of the high-CO_2_ seedlings was sufficient to produce higher biomasses than the ambient-CO_2_ plants during the entire pre-flowering development.

After transfer of the seedlings from agar to the 15-L sand culture, a third wave of increasing and decreasing RGRs was observed for both sets of plants between day 15 and day 21 after sowing (Fig. 4C). The maximum RGR of the high-CO_2_ plantlets exceeded that of the plantlets grown under ambient CO_2_ until day 22 while it was slightly lower during the following 2 weeks. From day 35 to day 61, the RGRs of plants of both sets were identical but decreased slightly.

Taken together, absolute biomass production was consistently higher under elevated than under ambient CO_2_. However, the major effects of elevated CO_2_ on RGR were observed during unfolding of the cotyledons and after transfer of the seedlings from the agar to the sand culture.

### Biomass allocation within the shoot and morphological characterization of tobacco plants grown at ambient or elevated CO_2_ concentrations

As shown before, the effect of elevated CO_2_ on growth and biomass production differed with the developmental stages of the plants. In order to examine the morphological differences of plants grown at ambient or elevated CO_2_ in more detail, the leaf number and leaf size of the plants (grown in 15-L pots) were examined at the ages of 28, 35, 42 and 49 days (Table 1). Plants grown under elevated CO_2_ concentration developed faster until the age of 42 days since they had one leaf more than those under ambient CO_2_. Elevated CO_2_ also stimulated the expansion of individual leaves (Table 1). However, the final leaf number at the emergence of flower buds was not increased by elevated CO_2_: at the age of 49 days, plants under both growth conditions had an equal leaf number.

**Figure 5.**
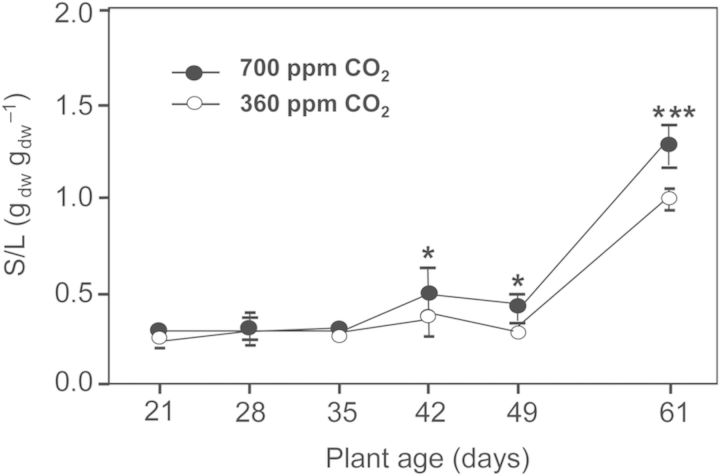
Stem:leaves ratios (St/L) of tobacco plants grown in 15-L sand culture under ambient (360 ppm) and elevated (700 ppm) CO_2_ concentration at different plant ages (means of *n* = 5 and standard deviation). Asterisks as in Fig. 2.

Biomass allocation to the stem and leaves, expressed as the stem/:leaves ratio (St/L in Fig. 5), was initially identical in high-CO_2_ and ambient-CO_2_ plants (Fig. 5). From 42 days on, the high-CO_2_ plants allocated more biomass to the stem than the ambient-CO_2_ plants. These differences were significant and increased slightly with plant age.

### Influence of CO_2_ concentration on the nutrient status of tobacco leaves

**Figure 6.**
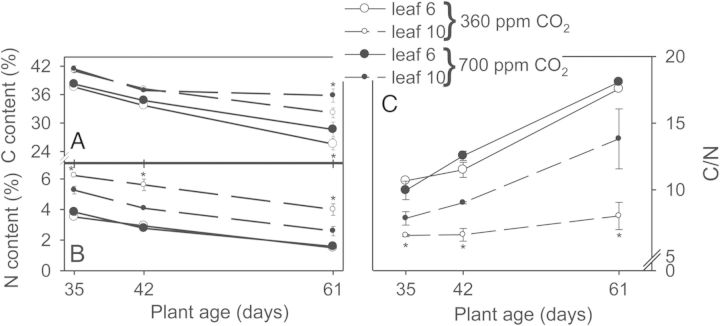
Age-dependent change of (A) carbon and (B) nitrogen content (%), and (C) C/N ratio of leaf 6 and leaf 10 of tobacco plants grown in 15-L sand culture under ambient (360 ppm) and elevated (700 ppm) CO_2_ concentrations (means of *n* = 3 and standard deviation). Asterisks as in Fig. 1.

The C/N ratios of two leaves each (leaves 6 and 10) were determined at the ages of 35, 42 and 61 days (Fig. 6). The contents of both carbon and nitrogen of all leaves decreased with age (Fig. 6A and B), but the carbon content of the EC leaves decreased less than that of the AC plants. An effect of the CO_2_ concentration on the nitrogen content was only observed in young leaves, the nitrogen content of which was lower in the high-CO_2_ plants. Since the age-dependent decrease of the portion of carbon was considerably larger than that of nitrogen in high-CO_2_ plants, the C/N ratio decreased substantially with increasing plant age. This decrease was less pronounced in ambient-CO_2_ plants (Fig. 6C).

## Discussion

In the present comprehensive study, the question of pseudoreplication must be addressed (see also the subsection Statistics): rather than duplicating the entire experimental setup, replicates were confined to the (great number of) samples. This appears permissible, as the results are interpreted to reflect the plants' response to an elevated but not to a particular CO_2_ concentration. In addition, growth conditions were carefully controlled during the entire experiment to minimize errors resulting from an unexpected environmental factor. Although the problem of pseudoreplication (*sensu* Hurlbert 1984) thus cannot be principally ruled out, the results are not at odds with the general view of the effects of an increasing CO_2_ atmosphere on the growth of plants. They provide, however, further insight into the role of nutrient acquisition in the response of the plant to an improved carbon source and in the signals involved in the adaptation of plants to the expected future environment.

Elevated CO_2_ concentration enhanced growth at all root-bed sizes by a factor of between 2.1 and 1.6, with a mean of 1.8. Root-bed volumes smaller than 15 L greatly inhibited biomass production irrespective of the CO_2_ concentration. When reviewing the effects of elevated CO_2_ on photosynthesis and biomass production of a variety of plants that were cultivated under controlled conditions in different root-bed volumes or in the field, Arp (1991) found a significant negative effect of a small root bed on both parameters, especially under high CO_2_. Small pots inhibit root growth and thus reduce its sink strength for biomass allocation. This effect was taken as evidence of a feedback regulation of a plant's photosynthetic rate by the demand of its sinks (Thomas and Strain 1991). On that background, root-bed restriction and elevated CO_2_ should result in an additive negative effect, as was observed by Arp (1991) as well as in our study by a stronger inhibition of biomass production at elevated than under ambient CO_2_ (steeper slope at elevated CO_2_ in Fig. 1D). Because our tobacco plants were grown from the very beginning, i.e. seed germination, under the two CO_2_ concentrations, the so-called acclimation at the transition from ambient to high CO_2_ could neither be expected nor was it observed during plant development (Fig. 4).

Our experiments showed that irrespective of the CO_2_ concentration, biomass allocation to the root increased with decreasing restriction of root growth by the pot (Fig. 1E). While this observation could be expected, another finding was at first glance surprising: the allocation of biomass to the root was higher under elevated than under ambient CO_2_. With one exception: the smallest pot, where the high-CO_2_ plants had a higher shoot-to-root biomass ratio. A lower ratio of shoot-to-root biomass in high-CO_2_ plants than in ambient-CO_2_ plants has been observed with a variety of plant species (for reviews, see Pritchard *et al.* 1999; Stitt and Krapp 1999; Yong *et al.* 2000; Yang *et al.* 2010; Wang *et al.* 2013) and appears to be a general phenomenon irrespective of root-bed size. Data from forested ecosystems revealed that elevated CO_2_ leads to an increased fine root production (Norby *et al.* 2004; Stiling *et al.* 2013) and to deeper rooting (for a review, see Iversen 2010). Consequently, the negative impact of a limiting root-bed size on root growth should be more pronounced at elevated CO_2_, which was indeed observed. But the increase of limitation under high CO_2_ was not as strong as supposed because the rates of net CO_2_ uptake of older, fully expanded leaves did not respond to the CO_2_ concentration **[see Supporting Information]**. A reason for that might be seen in a partial closure of the stomates of the high-CO_2_ leaves as a response to the elevated CO_2_ concentration. This explanation was corroborated by the transpiration rates of those leaves that were significantly higher under ambient than under high CO_2_ (Table 2). The stronger promotion of shoot than root growth of tobacco plants in 1-L pots at elevated CO_2_ was linked to a changed allocation pattern of the assimilates. One-litre pots were completely packed with roots, especially under elevated CO_2_. Root growth was more or less completely inhibited and the shoot received an excessive share of assimilates as indicated by the high C/N ratios of these plants (Fig. 2C).

A sequence of processes that take part in the regulation of growth have their origin in the uptake of macronutrients, in particular of nitrogen (McConnaughay *et al.* 1993; Stitt and Krapp 1999; Yang *et al.* 2007). Nutrient uptake is dependent on nutrient supply as well as on nutrient uptake associated with growth of the roots. Restriction of root growth results not only in an enhanced allocation of biomass to the root (decreasing S/R ratio) but also in a drop of the plant's nitrogen status (Ronchi *et al.* 2006; Yang *et al.* 2007). Both phenomena have been interpreted to reflect a decline of specific root functions in small pots (Yang *et al.* 2010). They were also observed in our experiments, in which the nitrogen content of the leaves increased with pot size (Fig. 2B). As expected, the nitrogen content of the youngest leaves (no. 20, as counted from the base) was much higher than that of leaves at the end of the expansion process (no. 10) or the oldest leaves at the bottom of the stem (no. 20). An age-dependent decline of the nitrogen content of the leaves is quite normal, and in tobacco the carbon content also decreases, due to the deposition of calcium oxalate as sand especially in the older leaves. The nitrogen contents of the leaves of plants grown at ambient CO_2_ were significantly higher than those of the leaves of high-CO_2_ plants irrespective of the position of the leaves. This finding reflects the increased demand for nutrients of the plants when grown under elevated CO_2_. Under that condition, the observed C/N ratios are understandable by assuming carbon limitation of growth under ambient CO_2_ and nitrogen limitation under enhanced CO_2_. Similar effects have been reported for a variety of other plant species (Yin 2002; for a review, see Taub and Wang 2008), and in more detail recently for wheat (Gutiérrez *et al.* 2013; Wang *et al.* 2013). Leaves of tobacco plants grown under high CO_2_ were consistently bigger than corresponding leaves of plants growing at ambient CO_2_ (Table 1), and if nutrient uptake by the roots does not match plant growth a reduced nutrient content must result. Another consequence of a reduced nutrient—especially nitrogen—availability under elevated CO_2_ is the enhanced formation of axial tissue, whose structural elements contain less nitrogen compared with leaf tissue (Fig. 5). In that context, it must be underlined that in contrast to all variants of nutrient supply described in the literature, our plants grew in purified quartz sand, which was continuously (day and night) flushed with a nutrient solution that had been optimized for the growth of tobacco. Therefore, restricted uptake rather than availability was the reason for nutrient limitation by the size of the root bed. Assuming the 15-L root-bed volume as sufficient for optimal root growth and maximal nutrient acquisition, the C/N ratios of plants grown in 15-L pots would reflect a kind of standard for optimal plant growth under the various CO_2_ concentrations. For the high-CO_2_ variant, this could even be the maximal achievable biomass production and growth of *N. tabacum* cv. *Samsun*.

### Cytokinins as potential signals in the realization of the effects of a restricted root bed and of growth under elevated CO_2_

Cytokinins as one group of phytohormones are associated with regulation of the size and activity of the shoot and root apical meristem (for a review, see Skylar and Wu 2011). The effective concentrations to promote root growth are low whereas shoot growth is favoured by relatively high concentrations (Skoog and Miller 1957; Cary *et al.* 1995; Werner *et al.* 2001, 2003, 2010).

Our experiments showed a clear positive correlation between root-bed size, cytokinin concentrations in the apices and developing leaves and growth of the shoot. They also showed, irrespective of root-bed volume, much lower concentrations in the roots than in the growth regions of the shoot. While cytokinin concentrations in the roots of plants from the 15-L pots were only slightly higher than those in the roots of corresponding plants from the 1-L pots, the concentration gradients between the roots and the shoots were much lower in the small plants from the 1-L pots. With *Urtica*, a close correspondence of the daily cytokinin export from the root to the shoot and the nutrient (nitrogen) status of the roots has been reported (Beck 1999), and molecular models for translation of the nitrogen status of the root into the cytokinin signal were presented (Takei *et al.* 2004; Sakakibara *et al.* 2006). However, a recent study with transgenic tobacco and *Arabidopsis* plants with a reduced cytokinin concentration in the roots showed stimulation of root growth by the lowered cytokinin concentration, but no change of the shoot phenotype. This was interpreted as an indication that the shoot growth is not directly controlled by the cytokinin supply from the roots. Instead, shoot meristems themselves seem to produce cytokinins in sufficient amounts to maintain growth (Werner *et al.* 2010).

At first glance, the identity of the cytokinin patterns and flow rates of the xylem fluids of ambient- and high-CO_2_ plants as measured at the base of the stems seems to corroborate the conclusions from studies with transgenic tobacco. In addition, the identity of the cytokinins in the xylem fluids of the petioles of mature leaves of plants grown under 360 and 700 ppm CO_2_, respectively, underlines this notion. In that case, however, the distinct differences in the qualitative and quantitative patterns of cytokinins between the xylem fluids of the stems and petioles require further explanation because both sampling positions were at most 10 cm apart. The fact that tobacco as a member of the *Solanaceae* family has a bicollateral vascular system with an interior and an exterior phloem might provide an explanation. Studies with tomato (Houngbossa and Bonnemain 1985) and *Nicotiana benthamiana* (Cheng *et al.* 2000) have suggested that the meristems of the shoots are supplied by the internal phloem, while export from source leaves to the roots is mainly by the external phloem. Thus the ‘xylem fluid’ collected at the bottom of the stem from an adequately pressurized root system is composed of true xylem fluid and the likewise upwards flowing content of the internal phloem. In contrast, xylem fluid collected from a petiole may only contain negligible amounts of (internal) phloem sap due to the comparably few internal phloem elements. The bulk of the content of the internal phloem of the shoot obviously bypasses the petioles of mature leaves and hence the exudates from the petiole rather reflect true xylem fluid. Transport from the external to the internal phloem via ray parenchyma cells is possible but slow as observed with transport of viruses (Cheng *et al.* 2000). It is also known that assimilates can move from the external phloem via stem ray cells to the xylem. The cytokinin content of that sap from high-CO_2_ plants was higher than from ambient-CO_2_ plants, irrespective of the shares at which the xylem and the internal phloem contributed to the exudates from pressurized roots. This finding could reflect the stronger root signal hypothesized for plants grown under elevated CO_2_ (Hypothesis iii; see also Yong *et al.* 2000). Because the patterns of the cytokinin species in the root exudates did not match those in the apical meristems or sink leaves, a direct contribution of the cytokinin root signal to the cytokinin content of the meristems is unlikely. Rather, this signal could trigger cell division associated with endogenous cytokinin production in the meristems.

### Elevated CO_2_ concentration already accelerated growth during germination and seedling development

Contrary to some reports in the literature (e.g. Miller *et al.* 1997; Ludewig and Sonnewald 2000), an accelerated ontogeny under elevated CO_2_ could only be observed at the very early stage when the cotyledons opened. They unfolded 1 day earlier under high than under ambient CO_2_. This advance turned out to be the basis for the persistently higher biomass production of plants grown at 700 ppm CO_2_ (Fig. 4B). Also, leaf formation was faster under high CO_2_, but the total leaf number per plant was equal under both CO_2_ concentrations until onset of flower bud formation (Table 1).

## Conclusions

Keeping several environmental factors constant, in particular nutrient concentration in the root bed, the effects of two variables, size of the root bed and atmospheric CO_2_ concentration, on the growth of tobacco plants could be compared. Elevated CO_2_ consistently stimulated growth but the effect of root-bed volume still overrode that effect (Hypothesis i). Limiting the production of new fine roots, spatial restriction of root growth in turn curtails nutrient uptake, as indicated by a higher C/N ratio of high-CO_2_ plants. Our experiments did not comprise a transfer of plants from an atmosphere of ambient CO_2_ into one with elevated CO_2_; thus the classical acclimation effect was not in the scope of this work. Nevertheless, higher RGRs could be observed during germination and seedling development, which after 3 weeks declined and subsequently equalled those of ambient-CO_2_ plants. In spite of this decrease in RGR, biomass production was consistently higher under elevated CO_2_ and thus acclimation did not take place (Hypothesis ii). The effect of root-bed volume was strongly mirrored by the cytokinin concentrations of the meristems of the shoot, but less so of the stem, mature leaves and roots. A similar but less pronounced effect on the cytokinin concentrations was seen from the CO_2_ concentration. In spite of the overall low cytokinin concentration in roots, the amounts of cytokinins moving from the root to the shoot were substantially higher in high-CO_2_ plants (Hypothesis iii). Part of this root signal most probably migrates via the internal phloem of the bicollateral vascular system of the tobacco plant. The composition of the cytokinin patterns appears to be one of the major control points in which various environmental cues are integrated into one signal for optimized growth of the (tobacco) plants.

## Sources of Funding

Our work was funded by the German Research Foundation with grant BE 473/23-4 to E.B.

## Contributions by the Authors

U.S. and B.D. conducted research, C.R. analysed data and wrote the manuscript, and E.B. designed the experiments, is the senior author and finalized the manuscript.

## Conflicts of Interest Statement

None declared.

## Supporting Information

The following **Supporting Information** is available in the online version of this article –

**Figure SI 1.** Regression lines resulting by plotting the products of length and width of the leaves of 42-day-old plants grown at 360 or 700 ppm CO_2_ against their areas determined with an area meter (means of *n* = 3 and standard deviations).

**Figure SI 2.** Concentrations of cytokinins in the xylem sap taken from the shoot base (representing the location of loading from the root into the shoot) or the petioles of source leaves (representing the location of unloading into the source leaf) of 35-day-old tobacco plants.

**Table SI 1.** Reactivities (‘cross-reactivities’) of the antibodies against DHZR, ZR and 2iPA with various cytokinin standards. The intensity of the reaction in ELISA with the immediate antigen was set at 100 %.

**Table SI 2.** Minimum amounts of fresh material used for cytokinin determination.

**Table SI 3.** CO_2_ net assimilation rates of a typical source (leaf no. 10) and a still growing leaf (leaf no. 15) of 42-day-old tobacco plants grown at ambient and 700 ppm CO_2_, respectively, in 15-L pots. Carbon dioxide gas exchange of the leaves was measured *in situ*. Measurements were performed with a portable porometer (HCM 1000; Heinz Walz GmbH, Effeltrich, Germany), which was placed in the climate cabinets. Since leaf no. 15 was ∼25 cm above leaf no. 10, it received a higher quantum flux density. The rates were means of five plants each with SE.

Additional Information
