# Supplementary material for: Influence of root-bed size on the response of tobacco to elevated CO2 as mediated by cytokinins
Source: AoB Plants. 2014 Mar 17;6:plu010. doi: 10.1093/aobpla/plu010 (PMC4038427; doi:10.1093/aobpla/plu010)
Supplement: Additional Information [file supp_6_plu010_index.html]

Influence of root-bed size on the response of tobacco to elevated CO2 as mediated by cytokinins — Additional Information 

# Influence of root-bed size on the response of tobacco to elevated CO2 as mediated by cytokinins

## Additional Information

Additional Information

**Files in this Data Supplement:**

- Additional Information - docx file
