# Supplementary material for: Influence of root-bed size on the response of tobacco to elevated CO2 as mediated by cytokinins
Source: AoB Plants. 2014 Mar 17;6:plu010. doi: 10.1093/aobpla/plu010 (PMC4038427; doi:10.1093/aobpla/plu010)
Supplement: Additional Information [file supp_plu010_plu010supp.docx]

**Supporting Information**

**Figure SI 1** Regression lines resulting by plotting the products of length and width of the leaves of 42 d old plants grown at 360 ppm or 700 ppm CO_2_ against their areas determined with an area meter (Delta-T Devices, Bunwell, Great Britain; means of n = 3 and standard deviations). The equations of the regression lines of the plants grown at 360 ppm or 700 ppm CO_2_ are identical. The factor 0.74 (r2 = 0.98) resulted from the regression line received when plotting the products of lengths and widths of 120 leaves of 6 plants against the leaf areas.

**Figure SI 2** Concentrations of cytokinins in the xylem sap of 35 d old tobacco plants taken from the shoot basis (representing the location of loading from the root into the xylem) or the petioles of source leaves (representing the location of unloading into the source leaf). Plants were cultivated at 360 ppm (□) or 700 ppm CO_2_ (■). Mean values of at least 2 independent experiments ± standard deviation are presented. Z(R)(N) – *t*-zeatin(riboside)(nucleotides) , DHZ(R)(N) – dihydrozeatin (riboside)(nucleotides), IP – isopentenyladenine, IPA – isopentenyladenosine, IPN – isopentenyl adenosine nucleotide.

**Table SI 1.** Reactivities („cross-reactivities“) of the antibodies against DHZR, ZR and 2iPA with various cytokinin standards. The intensity of the reaction in the ELISA with the immediate antigen was set at 100%.

| Cytokinine | Antibody against | | |
| --- | --- | --- | --- |
|  | DHZR | ZR | 2iPA |
| trans-ZR | 0.58 | 100.0000 | 0.21 |
| cis-ZR | 8.12 | 0.85 | 1.82 |
| Z | 0.17 | 44.500 | 0.08 |
| DHZR | 100.0000 | 2.77 | 0.19 |
| DHZ | 34.250 | 2.27 | 0.03 |
| 2iP | 0.80 | 0.28 | 58.940 |
| 2iPA | 1.14 | 0.28 | 100.0000 |
| Kinetin | 2.11 | 0.03 | 0.36 |
| Z9G | 2.57 | 87.300 | 1.43 |
| DHZ9G | 76.100 | 5.35 | 0.14 |
| Benzyladenosine | 4.11 | 0.92 | 12.130 |
| ZN | 2.42 | 64.360 | 0.25 |
| DHZN | 58.540 | 4.28 | 0.19 |
| 2iPN | 1.08 | 0.28 | 61.380 |
| ZROG | 0.02 | 0.25 | 00.002 |
| Z7G | 00.022 | 00.201 | 00.006 |

**Table SI 2.** Minimum amounts of fresh material used for cytokinin analysis (obtained also by pooling plant material: apices and sink leaves).

| Organ | Applied fresh material (g) | Number of plants |
| --- | --- | --- |
| Apex | 0.2 | 10 |
| Sink leaves | 0.3 | 3 |
| Stem | 10 | 1 |
| Source leaf | 15 | 1 |
| Root | 15 | 1 |

**Table SI 3.** CO_2_ net assimilation rates of a typical source (leaf no. 10) and a still growing leaf (leaf no. 15) of 42 d old tobacco plants grown at ambient and 700 ppm CO_2_, respectively in 15 l pots. CO_2_ gas exchange of the leaves was measured *in situ*. Measurements were performed with a portable Porometer (HCM 1000, Heinz Walz GmbH, Effeltrich, Germany) which was placed in the climate cabinets. Since leaf no. 15 was about 25 cm above leaf no. 10, it received a higher quantum flux density. The rates were means of 5 plants each with SE.

| CO_2_ concentration [ppm] | 360 | 700 |
| --- | --- | --- |
| Net CO_2_ uptake [µmol x m^-2^s^-1^] | | |
| Leaf Nr. 15 | 10.3 ± 2.2 | 12.6 ± 0.7 |
| Leaf Nr. 10 | 6.4 ± 0.2 | 6.4 ± 0.7 |
